# Patient experience of non-conveyance in the EMS of Southwest Finland: a descriptive survey study

**DOI:** 10.1186/s12873-024-00961-8

**Published:** 2024-03-13

**Authors:** Eetu Skaffari, Timo Iirola, Hilla Nordquist

**Affiliations:** 1 Centre for Prehospital Emergency Care, Emergency Medical Services, Wellbeing Services County of Pirkanmaa, Satakunnankatu 16, FI-33100 Tampere, Finland; 2 Emergency Medical Services, Turku University Hospital and University of Turku, FI-20521 Turku, PO Box 52, Finland; 3 Department of Healthcare and Emergency Care, South-Eastern Finland University of Applied Sciences, Pääskysentie 1, FI-48220 Kotka, Finland

**Keywords:** Emergency medical services (MeSH), Paramedics (MeSH), Surveys and questionnaires (MeSH), Patient experience, Non-conveyance

## Abstract

**Background:**

Emergency Medical Services are dispatched more frequently than before. However, many non-urgent patients do not need ambulance transportation to a healthcare facility after evaluation and treatment on scene. This study explored the experiences of non-conveyed patients. Our research questions were: (1) How have non-conveyed patients experienced the service received from EMS? (2) Does a patient’s age, gender, or time of the emergency call impact the patient’s experience?

**Methods:**

This descriptive survey study examined non-conveyed Emergency Medical Services patients in the Wellbeing Services County of Southwest Finland. The study period was from March 1, 2023, to March 31, 2023. The study population was 1017. They received a questionnaire that was sent by mail. The questionnaire was formed based on questions previously used in four different questionnaires. We received 247 answers (24.3% response rate). Percentages, medians with interquartile ranges, and non-parametric tests were used in the descriptive analyses.

**Results:**

Non-conveyed patients were very satisfied with the paramedics’ expertise and behavior, their ability to meet their individual needs, the sense of safety provided by the paramedics, and the instructions given to the patients. Time to receive help (19% rated 3 or less on a scale from 1 to 5), how paramedics introduced themselves (16.5%), and satisfaction with non-conveyance decisions (14.6%) were more frequently rated lower than other areas. Further, pain management stood out in the less favorable evaluations. Still, patients’ experiences of the service were positive. The age group, gender, or time of the emergency call were not associated with patient experience.

**Conclusions:**

Patients were very satisfied with the paramedics’ interpersonal skills. A more focused approach to pain management and developing EMS to ensure faster patient outreach and clearer explanations of non-conveyance decisions could further enhance the patient experience.

**Supplementary Information:**

The online version contains supplementary material available at 10.1186/s12873-024-00961-8.

## Background

Emergency Medical Services (EMS) are dispatched more often than before [1]. Elderly people use services the most. However, the incidence rate has grown in other age groups as well. There are other factors that explain the growth in demand, at least to some extent, such as the increase in mental health issues and substance abuse [2]. However, EMS seem to provide a larger amount of non-urgent healthcare [3], and many patients do not require transportation by ambulance to a healthcare facility after being assessed and treated at the scene by EMS [4–6]. Non-conveyance occurs in different types of EMS systems globally [6], and it is a safe practice as mortality in patients treated at scene is low [7]. Generally, the patients who do not need ambulance transportation tend to be younger than the ones who do [8].

While non-conveyance is a common practice in EMS, it is important to understand how patients experience the decision of not being transported to a hospital. Positive patient experience has been associated with higher levels of patient safety and clinical effectiveness, and, for example, in England, patient experience is reviewed as part of quality and performance reporting [9]. Understanding patient experience can be beneficial, as it enables service providers to gain insight into different aspects that patients value, which are not entirely measurable clinically, e.g., well-being and quality of life [10].

Many factors influence the patient experience in the decision of non-conveyance, such as the attitude and communication skills of the EMS personnel [11]. Generally, patients are satisfied with EMS personnel’s work and the care they receive, which they think is high quality. It increases the experience of safety when paramedics act calmly and empathically and if the ambulance arrives quickly [11, 12]. A Swedish study highlighted the importance of patient-centeredness and the everydayness of an EMS visit [13]. Patients in previous studies have experienced professionals treating them in a friendly manner and generally felt like they were treated as individuals and were given enough time by the paramedics [11, 12]. Patients and their family members have reported experiencing fear regarding an acute illness and felt reassured when the professionals were competent and thorough. In addition, providing self-care instructions or directing the patient to their own general practitioner made patients feel more confident about non-conveyance [11]. However, there is a difference in study results on whether the family members are less satisfied than the patients themselves [12, 14]. Another recent study from Sweden revealed that patients’ experiences of non-conveyance are versatile and complex, as they felt, for example, insecurity and uncertainty [15]. 

Patient experience should be of continual interest and in need of attention for the development of services. This topic is particularly relevant in the post-COVID-19 era, as healthcare services suffer from reduced work capacity and personnel shortages [16–18], which have been found to impact patient experience [19, 20]. Non-conveyance in EMS is still a relatively novel perspective in the study of patient experience and requires further investigation to improve the experience for all parties [21]. Previous evidence concerning non-conveyance from the perspective of patients or their significant others is mostly qualitative [11, 13, 14, 22]. This survey study enhances the understanding of the level of patient experience by offering all non-conveyed patients an opportunity to provide feedback on their experience during the research period. The perspectives and findings can be applied to the development of EMS services in contexts beyond Finland. The aim of this study was to explore the experiences of non-conveyed patients within the Wellbeing Services County of Southwest Finland. Our research questions were: (1) How have non-conveyed patients experienced the service received from EMS? (2) Does a patient’s age, gender, or time of the emergency call impact the patient’s experience?

## Materials and methods

We conducted a descriptive survey study on the non-conveyed EMS patients in Southwest Finland. The study period was from March 1, 2023, to March 31, 2023. Neither patients nor EMS personnel were informed of the study beforehand.

### Setting

In Finland, the statutory duties of EMS include triaging and treating patients in cases of injuries or acute sickness and conveying patients to the appropriate healthcare facility when needed. In the Finnish system, when dispatchers in the Emergency Response Centre process an emergency call and identify a need for healthcare services, they assign a mission to the EMS. These dispatchers are not healthcare professionals. The EMS is the sole healthcare provider dispatched through the Emergency Response Centre. This arrangement can partially lead to over-triage and emphasizes the importance of paramedics’ assessment in determining the necessity for care and making decisions regarding treatment and conveyance.

In Finland, Wellbeing Services Counties are responsible for organizing EMS. The Wellbeing Services County of Southwest Finland organizes social and health services and rescue operations in Southwest Finland, which has a population of 485,567 (31 December 2022) [23]. The area has 33 ambulances and a physician-staffed helicopter EMS unit. In addition, a helicopter unit of the Finnish Border Guard provides emergency care. There are approximately 65,000 EMS missions in the area annually.

In Southwest Finland, EMS units operate at an advanced level and are manned by two paramedics. At least one of the personnel needs to be an advanced level paramedic. Advanced level paramedics are registered nurses with an Emergency Care/Nursing dual bachelor’s degree or advanced-level out-of-hospital specialization. Basic level paramedics have a three-year vocational education. Firefighters can also work as basic level paramedics. Finnish legislation prescribes the required qualifications. Paramedics in Finland may always request care instructions from a physician..

### The questionnaire

The questionnaire was created by combining questions used for similar purposes in several previous studies. Some of the questions were rephrased from several questionnaires with similar questions, including Kuisma et al. (2003) (questions 6–11, 14, 16, 18, 19, and 24) [24], Johansson et al. (2011) (questions 12 and 13) [25], Marshall & Hays (1994) (questions 12 and 17) [26] and Bernard et al. (2007) (questions 12, 15, 21–23) [27] (Appendix 1). In addition, question 20 addressed patient satisfaction with the non-conveyance decision. Notably, not all questions are phrased in the same manner as in the original questionnaires. The questionnaire used in the study by Kuisma et al. (2003) [24] is mentioned as an example of a patient satisfaction questionnaire for EMS services in Finland’s Ministry of Social Affairs and Health publication (2019) [28]. This particular questionnaire in the mentioned publication has been updated from the original.

Five background questions were included. These were (1) who completed the questionnaire, (2) who made the emergency call, (3) what time, to the closest hour, the emergency call was made, (4) the patient’s gender, and (5) the patient’s age in years. The response options were the patient, relative, friend, or other (questions 1 and 2) and man, woman, other, or I don’t want to answer (question 4). Background questions 2, 4, and 5 and question 24 regarding the overall assessment of the service were featured in Finland’s Ministry of Social Affairs and Health questionnaire and publication (2019) [28]. The study group added questions 1 and 3.

Finally, the questionnaire consisted of the five mentioned background questions, 16 quantitative questions, and three additional qualitative questions. The results of the quantitative questions (1–20 and 24) are reported in this paper. A scale from 0 to 5 was used to measure patients’ experiences. On this scale, the options were described as very poor (1), poor (2), average (3), good (4), and excellent (5). 0 was used to describe questions that did not concern the respondent.

### The target group

During the study period, there were 1,312 EMS missions where the patient was not conveyed. The first author received a list (Microsoft Excel file) of all these missions, including the following data: date and time of dispatch, name, home address, information if the patient was a minor, and mission endpoint code (one of 10 different codes that the EMS unit send back to the dispatch centre to describe the reasons for non-conveyance).

The first author then removed duplicates from the data, and subsequently, all patients that did not meet the inclusion criteria were removed. The inclusion criteria were: (1) the person was a patient of EMS services in the study area between 1 March and 31 March 2023, (2) aged over 18 years old, (3) known home address in Finland and not in a long-term care facility, and (4) after examination and assessment by the paramedics or given treatment at scene the patient did not require a visit to the emergency department.

A total of 1,017 patients met the inclusion criteria of this study. The patients were sent an invitation letter with a questionnaire and information about data protection. Additionally, they were provided with a prepaid return envelope. Answering via the Webropol platform was also possible as the invitation letter contained a QR code leading to the online questionnaire.

### Statistical methods

The background information of the respondents is reported with percentages and frequencies. Due to a skewed distribution, age is reported with median (md) and interquartile range (IQR).

The responses under the topics of informing (Appendix 1, questions 11 and 12, Cronbach’s alpha 0.886), behavior (questions 13, 14 and 15, Cronbach’s alpha 0.943) and feeling of safety (questions 16 and 17, Cronbach’s alpha 0.918) were formed into composite variables by summing them up and then recalibrating the responses back to the original 1–5 scale. This was done by dividing the summed responses by the number of questions in the composite variable.

The distribution of all the responses is presented with percentages. The differences between age groups (≤ 64, 65–79, and ≥ 80 years old were formed based on the age classifications used in national population studies [29]), the time of the emergency call (daytime 8 am to 9 pm and nighttime 10 pm to 7 am), and women and men (there were too few individuals of other genders) were tested using Kruskal-Wallis and Mann-Whitney U tests. The significance level was p < 0.05. We reported the results by these groups using md and IQR, as our questionnaire had an ordinal scale of 1–5. Additionally, mean and standard deviation (sd) are also reported, following the example of previous studies [24–26]. The analyses were performed using SPSS version 28.

## Results

We received 247 answers (24.3% response rate) via post and online. Most of the respondents (61.5%) were women. The median age was 74, with an IQR of 60.5, 82.0. The background information of the respondents is described in Table 1.

The emergency call was usually made by the patient (43.3%) or a relative (40.5%). Most of the questionnaires were answered by the patients themselves (81%).

**Table 1:** Background information, n = 247

	%	n
**Age, years**		
≤ 64	30.0	74
65–79	36.8	91
≥ 80	32.4	80
Missing	0.8	2
**Gender**		
Woman	61.5	152
Man	38.1	94
Other	0.4	1
**Time of the emergency call**		
Daytime (8 am– 9 pm)	32.4	80
Nighttime (10 pm– 7 am)	49.0	121
Missing	18.6	46

**Fig. 1:**
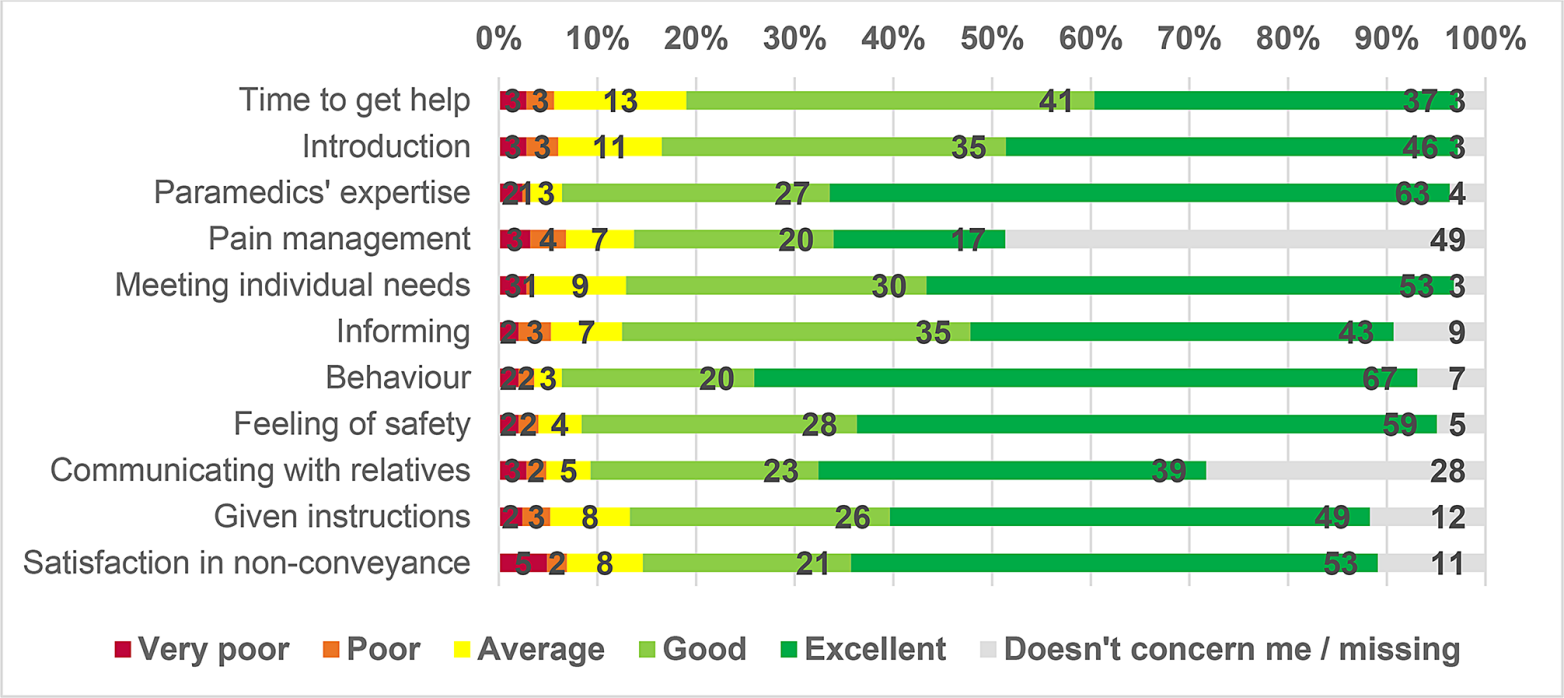
Distribution of answers (n = 247)

The paramedics’ behavior received the highest proportion of excellent evaluations (Fig. 1). The age group (Table 2), gender (Appendix 2), or time of the emergency call (Appendix 3) were not significantly related to patient experience. Respondents from all age groups gave the highest evaluations, with median values of 5 (excellent) and an interquartile range of 4, 5 to paramedics’ expertise, meeting individual needs, behavior, feeling of safety, given instructions, and satisfaction in non-conveyance (Table 2).

Pain management received the lowest evaluation, with the widest interquartile range observed among the age groups ≤ 64 years (median 4, IQR 3, 5) and 65–79 years (median 4, IQR 3, 5) (Table 2), although the results are still considered as good. When examining the distribution of the grades of very poor (1), poor (2) or average (3), lower evaluations were also given on time to get help (19% answered 3 or less), the way paramedics introduced themselves (16.5%) and satisfaction in non-conveyance (14.6%) (Fig. 1). Additionally, paramedics’ expertise and behavior both received evaluations of 3 or lower in only 6.4% of the responses.

**Table 2:** Descriptive results by age group (n = 247)

	Age ≤ 64		Age 65–79		Age ≥ 80		
	Md (IQR)	Mean (sd)	Md (IQR)	Mean (sd)	Md (IQR)	Mean (sd)	p-value*
Time to get help	4 (4, 5)	4.19 (1.04)	4 (4, 5)	4.11 (0.89)	4 (4, 5)	4.04 (0.86)	0.14
Introduction	5 (4, 5)	4.26 (0.96)	4 (4, 5)	4.23 (0.95)	4 (4, 5)	4.18 (0.93)	0.55
Paramedics’ expertise	5 (4,5)	4.52 (0.75)	5 (4, 5)	4.5 (0.88)	5 (4, 5)	4.64 (0.65)	0.60
Pain management	4 (3, 5)	4.00 (1.12)	4 (3, 5)	3.74 (1.20)	4 (4, 5)	3.97 (1.04)	0.97
Meeting individual needs	5 (4, 5)	4.28 (0.93)	5 (4, 5)	4.4 (0.93)	5 (4, 5)	4.43 (0.77)	0.31
Informing	4,5 (4, 5)	4.41 (0.75)	5 (4, 5)	4.39 (0.91)	4.5 (4, 5)	4.38 (0.75)	0.56
Behavior	5 (4.7, 5)	4.64 (0.69)	5 (4, 5)	4.51 (0.88)	5 (4.1, 5)	4.62 (0.66)	0.10
Feeling of safety	5 (4.4, 5)	4.57(0.766)	5 (4, 5)	4.49 (0.82)	5 (4, 5)	4.58 (0.74)	0.50
Communicating with relatives	4 (4, 5)	4.19 (1.09)	5 (4, 5)	4.29 (1.02)	5 (4, 5)	4.45 (0.87)	0.63
Given instructions	5 (4, 5)	4.19 (0.86)	5 (4, 5)	4.24 (1.09)	5 (4, 5)	4.35 (0.81)	0.28
Satisfaction in non-conveyance	5 (4, 5)	4.29 (1.16)	5 (4, 5)	4.33 (1.05)	5 (4, 5)	4.27 (1.08)	0.56

*Kruskall-Wallis test

Regarding the question of the overall assessment of the service (question 24, Appendix 1), most patients (41.3%) gave the highest grade of 5. Only 14 patients (5.7%) gave the lowest grade, meaning the received service had not helped them at all.

## Discussion

This study described the experience of 247 non-conveyed Emergency Medical Services patients in Southwest Finland. According to the main findings, patients expressed satisfaction with the paramedics’ expertise and behavior, their ability to meet individual needs, the feeling of safety provided, and the instructions given. The patient experience could be further enhanced in the areas of time to receive help, how paramedics introduced themselves, and pain management. Additionally, satisfaction with non-conveyance decisions had mixed responses, indicating room for improvement. However, all results indicated good patient experience. Patients’ age group, gender, or time of the emergency call were not significantly associated with patient experience.

There are many reasons why patients with non-urgent healthcare needs decide to contact EMS, including emotional factors, such as feelings of uncertainty and the need for reassurance [30]. Rantala et al. (2017) found that non-conveyed patients find the atmosphere during EMS missions to be patient-centered and highly value the feeling of safety [13]. This is in accordance with our finding that patients generally feel safe when in contact with paramedics. Our study also showed that patients in the study area are highly satisfied with paramedics’ expertise and behavior. Further, keeping the patient informed and giving instructions also received good evaluations, highlighting previous knowledge of shared decision-making and paramedics’ interpersonal skills in patient experience [11]. Paramedics in our study succeeded in meeting the patient’s individual needs.

In this study, not all patients were satisfied with the time it took to get help. In the study area, 90% of low urgency patients were reached within 1 h 37 min of dispatch during the period of January– March 2023 (chief EMS physician Iirola, T., personal communication [22 September 2023]). Generally, in Finland, paramedics should reach low-urgency patients triaged by the Emergency Response Centre within two hours. Patients who are triaged as more urgently in need of care receive help faster. A doctoral dissertation studied Finnish EMS and found that EMS response times were better when dispatched more urgently, although the response times vary between different EMS areas [31]. It is, of course, quite understandable that patients’ experiences regarding the waiting time vary. Even though the healthcare system sees a two-hour wait as an acceptable waiting time, it can still feel like an unacceptable long time for a patient in need of help.

It has been shown that pain is an under-recognized and under-treated symptom in EMS. Patients’ experiences of pain are often left unassessed [32]. Pain is still treated insufficiently even though adequate analgesia has a positive effect on patients [33]. Almost half of our study respondents did not feel this question concerned them. Although almost two out of five gave a good or excellent grade in pain management, approximately one out of seven gave a poorer (grade 3 or less) assessment. Likely, these results could still be improved. Future studies could determine how to treat pain more sufficiently; for example, do patients lack medical analgesia, or should EMS concentrate on pain management without drugs?

A previous study [34] revealed that patients do not always understand that an ambulance is a facility for treatment rather than a transportation method to a hospital. In this study, satisfaction with non-conveyance was one of the subjects that received mixed responses. As shown in previous studies regarding non-conveyance, many patients do not need ambulance transportation [5, 6], but they may want it [30]. This might imply that informing the public about the nature of EMS could further improve non-conveyance satisfaction. This has also been recommended by another study from the Netherlands [11]. It could be beneficial to study whether this kind of information campaign would result in higher satisfaction. However, most of the non-conveyed patients in our study thought that the service they received helped them, and the overall evaluations were good. These results are in accordance with previous Swedish, Australian and Finnish studies [12, 14, 21].

As previously mentioned, EMS operates at an advanced level in the study area. A Swedish study found that advanced level paramedics deliver high-quality care that patients value. Assessing and treating patients at the pre-hospital stage helps avoid unnecessary ambulance transports, which, for example, reduces discomfort [35]. Finnish paramedics’ high level of education could partly explain why patients were so satisfied with the care they received. However, further research is needed to determine what kind of education on interpersonal skills would be beneficial to improve patient experience. Further, these study results could be used to develop paramedics’ basic education.

### Limitations of the study

A strength of the data collection in this study is that the EMS of the studied area uses electronic patient records. Thus, we received a comprehensive document including all the needed information to include or exclude patients in this study. The questionnaire (Appendix 1) was formed based on several previously used questionnaires. The research team with significant work experience in healthcare and scientific research selected the questions. Additionally, we aimed to closely adhere to prior studies by retaining the original question formulations. This could have led to limitations; for instance, the expertise question [24] includes two distinct tasks– the ability to carry out procedures, which is more practical, and recognizing patients’ problems, demanding more theoretical knowledge. This dual nature may compromise the reliability of the results.

Before data collection, the questionnaire was presented for feedback to a group of advanced level paramedics from different parts of Finland. Their feedback was valuable in refining the questions further. This contributes to the reliability of the questionnaire. However, to improve the questionnaire, it could have been tested on a group of patients to receive feedback from the target group. This would have ensured that the questionnaire accurately addressed their experiences and concerns.

Our study was based on a convenience sample, and we did not conduct power calculations for each group studied (age, gender, time of the emergency call). This approach was considered appropriate given the descriptive nature of our study, and detailed demographic data of the target group were not available to the research team during the study’s planning phase. Further, we did not have the full age and gender distribution of the target group, preventing a complete representative comparison. Our response rate was 24.3%. Due to the uniformity of the answers received, the material was considered reliable and applicable for statistical testing. Still, the possibility of sampling bias should be noted when interpreting the results.

Another limitation is that there are dozens of long-term care facilities in the study area, and we did not have access to a list of these. Therefore, it is possible that some of the letters were addressed to patients living in facilities. Including patients living in long-term care facilities could have influenced the results obtained. However, they were excluded because of their potential inability to respond to the survey themselves due to health conditions (e.g., dementia). Another clear limitation is that the letter was only in Finnish. The study group considered translating the letter into Swedish, but this was not done due to the weight limit of the letter. According to Statistics Finland, on 31.12.2022, 5.7% of people in the Wellbeing Services County of Southwest Finland spoke Swedish as their native language. 89 persons per 1,000 inhabitants spoke languages other than Finnish, Swedish, or Sami [36]. Some of the questionnaires were answered by someone other than the patient. Although the questionnaire asked for the patients’ experiences, it is possible that the answers do not represent patients’ opinions if another person filled in the questionnaire.

This study provides a basis for subsequent quantitative research on patient experience. Our response rate of 24.3% can be considered to provide good external validity, thereby allowing the results to be generalized to the entire non-conveyance patient group of Southwest Finland. In addition, the results can be generalized with caution to other areas in Finland and are useful to global EMS systems similar to the Finnish system. Differences in systems, such as a lack of consultation possibilities or differences in the paramedics’ educational level and competence, may weaken the generalizability of our results.

## Conclusions

Generally, the patients of the EMS in Southwest Finland are satisfied with the service they receive when evaluated and treated at the scene. Non-conveyed patients were very satisfied with the paramedics’ expertise and behavior, their ability to meet the patient’s individual needs, the sense of safety provided by the paramedics, and the instructions given to the patients. The patient experience could be improved by reaching patients faster, remembering to make proper introductions, and addressing the concerns raised with pain management. Justifications for non-conveyance decisions may also require further clarification and improved communication with the patient and their significant other(s). Patient experience regarding non-conveyance would benefit from further research conducted in different areas and EMS systems to continue developing paramedics’ education and improving the service’s relevance and efficacy.

### Electronic supplementary material

Below is the link to the electronic supplementary material.

Supplementary Material 1

Supplementary Material 2

Supplementary Material 3

## Data Availability

The datasets generated and analyzed during the current study are not publicly available due to the limitations of the research permit but are available from the corresponding author upon reasonable request.

## References

[1] Christensen EF, Bendtsen MD, Larsen TM, Jensen FB, Lindskou TA, Holdgaard HO, Hansen PA, Johnsen SP, Christiansen CF (2017). Trends in diagnostic patterns and mortality in emergency ambulance service patients in 2007–2014: a population-based cohort study from the North Denmark Region. BMJ Open.

[2] Andew E, Nehme Z, Cameron P, Smith K (2019). Drivers of increasing emergency ambulance demand. Prehosp Emerg Care.

[3] Pekanoja S, Hoikka M, Kyngäs H, Elo S (2018). Non-transport emergency medical service missions– a retrospective study based on medical charts. Acta Anaesthesiol Scand.

[4] Magnusson C, Herlitz J, Axelsson C (2020). Patient characteristics, triage utilization, level of care, and outcomes in an unselected adult patient population seen by the emergency medical services: a prospective observational study. BMC Emerg Med.

[5] Ebben RH, Vloet LC, Speijers RF, Tönjes NW, Loef J, Pelgrim T, Hoogeveen M, Berben SA (2017). A patient-safety and professional perspective on non-conveyance in ambulance care: a systematic review. Scand J Trauma Resusc Emerg Med.

[6] Paulin J, Kurola J, Salanterä S, Moen H, Guragain N, Koivisto M, Käyhkö N, Aaltonen V, Iirola T (2020). Changing role of EMS– analyses of non-conveyed and conveyed patients in Finland. Scand J Trauma Resusc Emerg Med.

[7] Paulin J, Kurola J, Koivisto M, Iirola T (2021). EMS non-conveyance: a safe practice to decrease ED crowding or a threat to patient safety?. BMC Emerg Med.

[8] Lederman J, Lindström V, Elmqvist C, Löfvenmark C, Djärv T (2020). Non-conveyance in the ambulance service: a population-based cohort study in Stockholm, Sweden. BMJ Open.

[9] Gleeson H, Calderon A, Swami V, Deighton J, Wolpert M, Edbrooke-Childs J (2016). Systematic review of approaches to using patient experience data for quality improvement in healthcare settings. BMJ Open.

[10] LaVela SL, Gallan AS (2014). Evaluation and measurement of patient experience. Patient Experience J.

[11] Van Doorn SC, Verhalle RC, Ebben RH, Frost DM, Vloet LC, De Brouwer CP (2021). The experience of non-convoyance following emergency medical service triage from the perspective of patients and their relatives: a qualitative study. Int Emerg Nurs.

[12] Salminen-Tuomaala M, Mikkola R, Paavilainen E, Leikkola P (2018). Emergency patents’ and family members’ experiences of encountering care providers and receiving care in nonconveyance situations. Scand J Caring Sci.

[13] Rantala A, Forsberg A, Ekwall A (2017). Person-centered climate and psychometrical exploration of person-centeredness among patients not conveyed by the Ambulance Care Service. Scand J Caring Sci.

[14] Larsson G, Dagerhem A, Wihlborg J, Rantala A (2022). Satisfaction among non-conveyed patients and significant others when discharged at the scene by the ambulance service: an exploratory cross-sectional survey. BMC Emerg Med.

[15] Lederman J, Löfvenmark C, Djärv T, Lindström V, Elmqvist C (2023). A phenomenological interview study with patients being non-conveyed in the ambulance service. BMC Emerg Med.

[16] King R, Oprescu F, Lord B, Flanagan B (2021). Patient experience of non-conveyance following emergency ambulance service response: a scoping review of the literature. Australas Emerg Care.

[17] Rantala A, Ekwall A, Forsberg A (2019). Significant others’ perceptions of being taken seriously by the Swedish ambulance service when the patient is assessed as nonurgent. Scand J Caring Sci.

[18] de Vries N, Boone A, Godderis L (2023). The race to Retain Healthcare Workers: a systematic review on factors that Impact Retention of Nurses and Physicians in hospitals. Inquiry.

[19] Chemali S, Mari-Sáez A, El Bcheraoui C, Weishaar H (2022). Health care workers’ experiences during the COVID-19 pandemic: a scoping review. Hum Resour Health.

[20] Lee BEC, Ling M, Boyd L, Olsson C, Sheen J (2023). The prevalence of probable mental health disorders among hospital healthcare workers during COVID-19: a systematic review and meta-analysis. J Affect Disord.

[21] Winter V, Schreyögg J, Thiel A (2020). Hospital staff shortages: environmental and organizational determinants and implications for patient satisfaction. Health Policy.

[22] Clark PA, Leddy K, Drain M, Kaldenberg D (2007). State nursing shortages and patient satisfaction: more RNs–better patient experiences. J Nurs Care Qual.

[23] Statistic Finland. StatFin / Population structure / 11ra -- Key figures on population by region, 1990–2022. https://pxdata.stat.fi/PxWeb/pxweb/en/StatFin/StatFin__vaerak/statfin_vaerak_pxt_11ra.px/table/tableViewLayout1/ Accessed 27 October 2023.

[24] Kuisma M, Määttä T, Hakala T, Sivula T, Nousila-Wiik M (2003). Customer satisfaction measurement in emergency medical services. Acad Emerg Med.

[25] Johansson A, Ekwall A, Wihlborg J (2011). Patient satisfaction with ambulance care services: survey from two districts in southern Sweden. Int Emerg Nurs.

[26] Marshall GN, Ron D (1994). Hays. The patient satisfaction questionnaire short-form (PSQ-18).

[27] Bernard AW, Lindsell CJ, Handel DA, et al. Postal survey methodology to assess patient satisfaction in a suburban emergency medical services system: an observational study. BMC Emerg Med. 2007;7:5. 10.1186/1471-227X-7-5. Published 2007 Jun 15. 10.1186/1471-227X-7-5 PMC1904228 17573969

[28] Kuisma M, Järvelin J, Kilpiäinen E, Tuukkonen J, Pöllänen R, Saarinen M, Vaula E, Wilen S, Etelälahti T. Quality and patient safety in emergency medical services and hospital emergency department services– from planning to implementation and evaluation. Ministry Social Affairs Health: Publications Ministry Social Affairs Health 2019:23. https://julkaisut.valtioneuvosto.fi/bitstream/handle/10024/161737/STM_2019_23_Laatu-_ja_potilasturvallisuus_ensihoidossa_ja_paivystyksessa.pdf (In Finnish.).

[29] Statistics Finland. StatFin / Population structure / 11ra -- Key figures on population by region, 1990–2022. https://pxdata.stat.fi/PxWeb/pxweb/en/StatFin/StatFin__vaerak/statfin_vaerak_pxt_11ra.px/ Accessed 12 Feb 2024.

[30] Booker MJ, Purdy S, Shaw ARG (2017). Seeking ambulance treatment for ‘primary care’ problems: a qualitative systematic review of patient, carer and professional perspectives. BMJ Open.

[31] Ilkka L. Emergency Medical Services (EMS) in Finland: National data management as a path to better prehospital care [dissertation]. University of Eastern Finland, Kuopio; 2022. Chapter 6.2.5, Availability of EMS to society; p. 210–211.

[32] Ferri P, Gambaretto C, Alberti S, Parogni P, Rovesti S, Di Lorenzo R, Sollami A, Bargellini A (2022). Pain Management in a Prehospital Emergency setting: a retrospective observational study. J Pain Res.

[33] Akbas S, Castellucci C, Nehls F, Müller SM, Spahn DR, Kaserer A (2022). Präklinische Schmertxtherapie: Übersicht Und Verbesserungsmöglichkeiten. Praxis.

[34] Ahl C, Nyström M (2012). To handle the unexpected– the meaning of caring in pre-hospital emergency care. Int Emerg Nurs.

[35] Jansson J, Larsson M, Nilsson J (2021). Advanced paramedics and nurses can deliver safe and effective prehospital and in-hospital emergency care: an integrative review. Nurs Open.

[36] Statistics Finland. StatFin / Population structure / 11rl -- Language according to age and sex by region, 1990–2022. https://pxdata.stat.fi/PxWeb/pxweb/en/StatFin/StatFin__vaerak/statfin_vaerak_pxt_11rl.px/table/tableViewLayout1/ Accessed 27 Oct 2023.

[37] Finnish National Board on Research Integrity TENK. 2023. The Finnish Code of Conduct for Research Integrity and Procedures for Handling Alleged Violations of Research Integrity in Finland. https://tenk.fi/sites/default/files/2023-05/RI_Guidelines_2023.pdf. Accessed 20 Sept 2023.

[38] Finnish National Board on Research and Integrity TENK. 2020. The ethical principles of research with human participants and ethical review in the human sciences in Finland. https://tenk.fi/sites/default/files/202101/Ethical_review_in_human_sciences_2020.pdf. Accessed 20 Sept 2023.

