# Supplementary material for: Patient experience of non-conveyance in the EMS of Southwest Finland: a descriptive survey study
Source: BMC Emerg Med. 2024 Mar 13;24:42. doi: 10.1186/s12873-024-00961-8 (PMC10935972; doi:10.1186/s12873-024-00961-8)
Supplement: Supplementary file 1 — Supplementary Material 1 [file 12873_2024_961_MOESM1_ESM.docx]

Appendix 3. Results by time of the emergency call.

|  | Daytime (8 am – 9 pm) | | | Nighttime (10 pm – 7 am) | | |  |
| --- | --- | --- | --- | --- | --- | --- | --- |
|  | n | Md (IQR) | Mean (sd) | n | Md (IQR) | Mean (sd) | p-value* |
| Time to get help | 80 | 4 (4, 5) | 4.18 (0.84) | 116 | 4 (4, 5) | 4.08 (0.95) | 0.60 |
| Introduction | 79 | 4 (4, 5) | 4.13 (0.94) | 116 | 5 (4, 5) | 4.32 (0.87) | 0.09 |
| Paramedics’ expertise | 80 | 5 (4, 5) | 4.56 (0.71) | 114 | 5 (4, 5) | 4.61 (0.63) | 0.81 |
| Pain management | 38 | 4 (3, 5) | 3.55 (1.29) | 61 | 4 (4, 5) | 4.05 (0.87) | 0.06 |
| Meeting individual needs | 79 | 5 (4, 5) | 4.33 (0.93) | 115 | 5 (4, 5) | 4.43 (0.76) | 0.61 |
| Informing | 77 | 4.5 (4, 5) | 4.38 (0.74) | 109 | 4.5 (4, 5) | 4.47 (0.69) | 0.45 |
| Behavior | 78 | 5 (4, 5) | 4.52 (0.79) | 110 | 5 (4.3, 5) | 4.67 (0.59) | 0.15 |
| Feeling of safety | 78 | 5 (4, 5) | 4.49 (0.86) | 114 | 5 (4.38, 5) | 4.62 (0.62) | 0.63 |
| Communicating with relatives | 63 | 5 (4, 5) | 4.30 (1.02) | 85 | 5 (4, 5) | 4.35 (0.87) | 0.94 |
| Given instructions | 74 | 5 (4, 5) | 4.32 (0.89) | 106 | 5 (4, 5) | 4.42 (0.79) | 0.47 |
| Satisfaction in non-conveyance | 74 | 5 (4, 5) | 4.12 (1.21) | 105 | 5 (4, 5) | 4.39 (0.98) | 0.15 |

* Mann-Whitney U test
