# Supplementary material for: Patient experience of non-conveyance in the EMS of Southwest Finland: a descriptive survey study
Source: BMC Emerg Med. 2024 Mar 13;24:42. doi: 10.1186/s12873-024-00961-8 (PMC10935972; doi:10.1186/s12873-024-00961-8)
Supplement: Supplementary file 2 — Supplementary Material 2 [file 12873_2024_961_MOESM2_ESM.docx]

**Appendix 1. Questionnaire**

3/4

4/4
