# Supplementary material for: Patient experience of non-conveyance in the EMS of Southwest Finland: a descriptive survey study
Source: BMC Emerg Med. 2024 Mar 13;24:42. doi: 10.1186/s12873-024-00961-8 (PMC10935972; doi:10.1186/s12873-024-00961-8)
Supplement: Supplementary file 3 — Supplementary Material 3 [file 12873_2024_961_MOESM3_ESM.docx]

Appendix 2. Results by gender.

|  | Woman | | | Man | | |  |
| --- | --- | --- | --- | --- | --- | --- | --- |
|  | n | Md (IQR) | Mean (sd) | n | Md (IQR) | Mean (sd) | p-value* |
| Time to get help | 148 | 4 (4, 5) | 4.16 (0.95) | 91 | 4 (4, 5) | 4.01 (0.94) | 0.14 |
| Introduction | 148 | 4 (4, 5) | 4.18 (1.00) | 91 | 4 (4, 5) | 4.27 (0.91) | 0.55 |
| Paramedics’ expertise | 149 | 5 (4, 5) | 4.50 (0.86) | 88 | 5 (4, 5) | 4.59 (0.71) | 0.60 |
| Pain management | 80 | 4 (3, 5) | 3.88 (1.15) | 47 | 4 (4, 5) | 3.87 (1.15) | 0.96 |
| Meeting individual needs | 147 | 5 (4, 5) | 4.32 (0.92) | 91 | 5 (4, 5) | 4.42 (0.88) | 0.31 |
| Informing | 140 | 4.5 (4, 5) | 4.34 (0.89) | 85 | 4.5 (4, 5) | 4.45 (0.74) | 0.56 |
| Behavior | 144 | 5 (4.1, 5) | 4.53 (0.81) | 85 | 5 (4.5, 5) | 4.64 (0.75) | 0.10 |
| Feeling of safety | 148 | 5 (4, 5) | 4.51 (0.84) | 87 | 5 (4, 5) | 4.57 (0.76) | 0.51 |
| Communicating with relatives | 104 | 5 (4, 5) | 4.34 (0.98) | 73 | 5 (4, 5) | 4.27 (1.01) | 0.63 |
| Given instructions | 134 | 5 (4, 5) | 4.35 (0.97) | 84 | 4.5 (4, 5) | 4.25 (0.96) | 0.28 |
| Satisfaction in non-conveyance | 134 | 5 (4, 5) | 4.30 (1.05) | 85 | 5 (4, 5) | 4.31 (1.16) | 0.58 |

* Mann-Whitney U test
